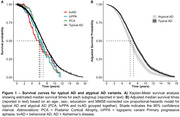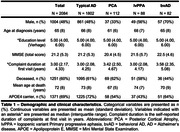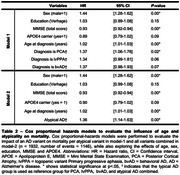# An atypical clinical phenotype is a risk factor for higher mortality in Alzheimer’s disease

**DOI:** 10.1002/alz.087308

**Published:** 2025-01-09

**Authors:** Ilse Bader, Colin Groot, Wiesje M. van der Flier, Yolande A.L. Pijnenburg, Rik Ossenkoppele

**Affiliations:** ^1^ Amsterdam Neuroscience, Neurodegeneration, Amsterdam Netherlands; ^2^ Alzheimer Center Amsterdam, Neurology, Vrije Universiteit Amsterdam, Amsterdam UMC location VUmc, Amsterdam Netherlands; ^3^ Lund University, Clinical Memory Research Unit, Lund Sweden; ^4^ Epidemiology and Data Science, Vrije Universiteit Amsterdam, Amsterdam UMC location VUmc, Amsterdam, Noord‐Holland Netherlands; ^5^ Alzheimer Center Amsterdam, Neurology, Vrije Universiteit Amsterdam, Amsterdam UMC, Amsterdam Netherlands; ^6^ Amsterdam Neuroscience, Neurodegeneration, Amsterdam, Noord‐Holland Netherlands

## Abstract

**Background:**

Survival estimates for individuals with Alzheimer’s disease (AD) are informative to understand the full disease trajectory. A previous meta‐analysis estimated the mean survival of AD patients at 5.8 years from diagnosis, but precise estimates for atypical AD variants are scarce. Atypical AD variants are characterized by a non‐amnestic phenotype, a relatively early‐onset and lower prevalence of APOE4, which are all possible modulators of clinical trajectories. Here we aimed to evaluate how phenotypical atypicality affects mortality for posterior cortical atrophy (PCA; visual‐AD), logopenic variant primary progressive aphasia (lvPPA; language‐AD) and behavioral‐AD (bvAD) versus a typical AD reference group while taking other risk‐modulators into account.

**Method:**

2084 amyloid‐positive cases from the Amsterdam Dementia Cohort were grouped into typical AD (n = 1802) versus atypical AD (n = 282; PCA [n = 112], lvPPA [n = 88], and bvAD [n = 82]) cases. Kaplan‐Meier analysis was performed to obtain survival estimates from diagnosis to death. Cox proportional‐hazard models were performed to assess effects of atypical AD on mortality, using typical AD as the reference group. We modeled atypical AD subtypes separately (model‐1) or combined (model‐2) while accounting for the potential effects of age, sex, education, MMSE and APOE4 on mortality. A Likelihood‐ratio test was performed to assess whether model‐2 with atypical AD provided a better model‐fit than model‐2 without atypical AD.

**Result:**

Table‐1 provides sample characteristics. Median survival times were 6.3[95%CI:5.7‐7.6] for lvPPA and 6.0[5.4‐6.9] for PCA, compared to 7.0[6.7‐7.2] for typical AD. The median survival time for bvAD 5.9[4.3‐9.0] could only be extrapolated given that 44% had deceased (Figure‐1a). Adjusted median survival times were 7.0[6.7‐7.2] for typical and 6.1[5.7‐6.5] for atypical AD as a combined group (Figure‐1b). In model‐1, PCA (HR = 1.37[1.06‐1.78]), male sex (HR = 1.44[1.28‐1.62]), age (HR = 1.02[1.01‐1.03]), and lower MMSE (HR = 0.93[0.92‐0.94]) were associated with increased mortality risk (Table‐2). All atypical AD variants combined were associated with increased mortality risk (HR = 1.36[1.14‐1.63]) compared to typical AD, and addition of atypical AD to the model significantly improved the model‐fit (X^2^ = 10.5;p<0.01).

**Conclusion:**

Survival in atypical AD is shorter compared to typical AD, even when correcting for other risk‐modulators. Hence, atypicality is an important risk factor for mortality beyond age, sex, education and MMSE.